# Apparent diffusion coefficient for molecular subtyping of non-gadolinium-enhancing WHO grade II/III glioma: volumetric segmentation versus two-dimensional region of interest analysis

**DOI:** 10.1007/s00330-018-5351-0

**Published:** 2018-03-23

**Authors:** S. C. Thust, S. Hassanein, S. Bisdas, J. H. Rees, H. Hyare, J. A. Maynard, S. Brandner, C. Tur, H. R. Jäger, T. A. Yousry, L. Mancini

**Affiliations:** 1 Neuroradiological Academic Unit, Department of Brain Repair and Rehabilitation, UCL Institute of Neurology, London, UK; 2 Imaging Department, University College London Foundation Hospital, London, UK; 3 Lysholm Department of Neuroradiology, National Hospital for Neurology and Neurosurgery, Queen Square, London, WC1N 3BG UK; 4 Department of Clinical Neurology, National Hospital for Neurology and Neurosurgery, London, UK; 5 Department of Neurodegenerative Disease, UCL Institute of Neurology and Division of Neuropathology, London, UK; 6 Queen Square MS Centre. Department of Neuroinflammation, UCL Institute of Neurology, University College London, London, UK

**Keywords:** Brain, Diffusion magnetic resonance imaging, Isocitrate dehydrogenase, Glioma, Neuroimaging

## Abstract

**Objectives:**

To investigate if quantitative apparent diffusion coefficient (ADC) measurements can predict genetic subtypes of non-gadolinium-enhancing gliomas, comparing whole tumour against single slice analysis.

**Methods:**

Volumetric T2-derived masks of 44 gliomas were co-registered to ADC maps with ADC mean (ADC_mean_) calculated. For the slice analysis, two observers placed regions of interest in the largest tumour cross-section. The ratio (ADC_ratio_) between ADC_mean_ in the tumour and normal appearing white matter was calculated for both methods.

**Results:**

Isocitrate dehydrogenase (IDH) wild-type gliomas showed the lowest ADC values throughout (*p* < 0.001). ADC_mean_ in the IDH-mutant 1p19q intact group was significantly higher than in the IDH-mutant 1p19q co-deleted group (*p* < 0.01). A volumetric ADC_mean_ threshold of 1201 × 10^−6^ mm^2^/s identified IDH wild-type with a sensitivity of 83% and a specificity of 86%; a volumetric ADC_ratio_ cut-off value of 1.65 provided a sensitivity of 80% and a specificity of 92% (area under the curve (AUC) 0.9–0.94). A slice ADC_ratio_ threshold for observer 1 (observer 2) of 1.76 (1.83) provided a sensitivity of 80% (86%), specificity of 91% (100%) and AUC of 0.95 (0.96). The intraclass correlation coefficient was excellent (0.98).

**Conclusions:**

ADC measurements can support the distinction of glioma subtypes. Volumetric and two-dimensional measurements yielded similar results in this study.

**Key Points:**

*• Diffusion-weighted MRI aids the identification of non-gadolinium-enhancing malignant gliomas*

*• ADC measurements may permit non-gadolinium-enhancing glioma molecular subtyping*

*• IDH wild-type gliomas have lower ADC values than IDH-mutant tumours*

*• Single cross-section and volumetric ADC measurements yielded comparable results in this study*

## Introduction

Gadolinium contrast uptake was previously considered the best MR imaging predictor of glioma histological grade and malignancy[1–3]. On the basis of this, it has been common practice to interpret non-enhancing intrinsic tumours as probable low grade gliomas (LGG) [4]. But conventional MRI has proven to be unreliable in predicting subsequent tumour behaviour, whereby a proportion of presumed LGG may rapidly progress with development of malignant features such as enhancement and necrosis [4–8].

The discovery of several key genetic alterations as principal determinants of glioma prognosis has challenged the reference standard of glioma grouping by histology [9]. Mutations in isocitrate dehydrogenase (IDH) represent a common (> 70%) defining event in the development of LGG, conversely more than 90% of glioblastomas belong to the IDH wild-type group [10, 11]. Despite its oncogenic effect through production of a toxic metabolite D2-hydroxyglutarate (2HG), the presence of an IDH mutation is associated with a favourable prognosis.

The revised 2016 World Health Organization (WHO) classification of brain tumours for the first time incorporates molecular data to augment the diagnosis [12]. For WHO grade II/III gliomas, three molecular subgroups have been defined: IDH wild-type glioma (IDH^wt^) with survival similar to that of glioblastoma, IDH-mutant glioma with intact 1p19q (IDH^mut^1p19^int^) and an intermediate prognosis, and IDH-mutant 1p19q co-deleted glioma (IDH^mut^1p19q^del^) with the best prognosis and greatest chemosensitivity [11]. There is partial overlap with histomorphology, whereby many IDH^mut^1p19^int^ are astrocytic and the majority of IDH^mut^1p19q^del^ belong to the oligodendroglioma group [13]. IDH^wt^ gliomas probably constitute a genetically heterogeneous category of lesions, but often exhibit aggressive behaviour and have been suspected to represent early glioblastoma [14–17]. In the emerging literature on MR imaging features of IDH^wt^ glioma, initial lack of enhancement has been reported in some of these tumours [6, 18, 19].

Diffusion-weighted imaging (DWI) is a technique of great interest in cancer, because water diffusivity is impaired in highly cellular tissues, which reflects tumour proliferative rate and aggressiveness [20]. The phenomenon of reduced diffusion preceding fulminant radiological progression of presumed LGG has been observed prior to molecular typing [7], evoking later descriptions of IDH^wt^ glioma serial imaging findings [4]. Quantitative apparent diffusion coefficient (ADC) values have demonstrated high accuracy for glioma grading through meta-analysis [21]. For the non-invasive identification of low to intermediate IDH^wt^ glioma, diffusion tensor imaging (DTI) and diffusion kurtosis imaging (DKI) have shown potential, suggesting that reduced and heterogenous diffusivity are IDH^wt^ features [22–24]. However, advanced diffusion techniques are not universally available outside academic hospital institutions, may require longer scan times and dedicated post-processing.

Mean ADC measurement could be a rapid and practicable approach to assess glioma diffusivity, being computationally non-demanding compared to histograms or texture analysis. Although theoretically superior, there is no conclusive evidence that whole lesion analysis outperforms region-of-interest placement for the identification of malignant gliomas [25].

The study presented sought to (i) investigate whether ADC measurements from routine clinical DWI were associated with glioma molecular subtype and (ii) to compare the performance of volumetric whole tumour ADC with single slice ADC measurements.

## Materials and methods

### Patients

**Fig. 1:**
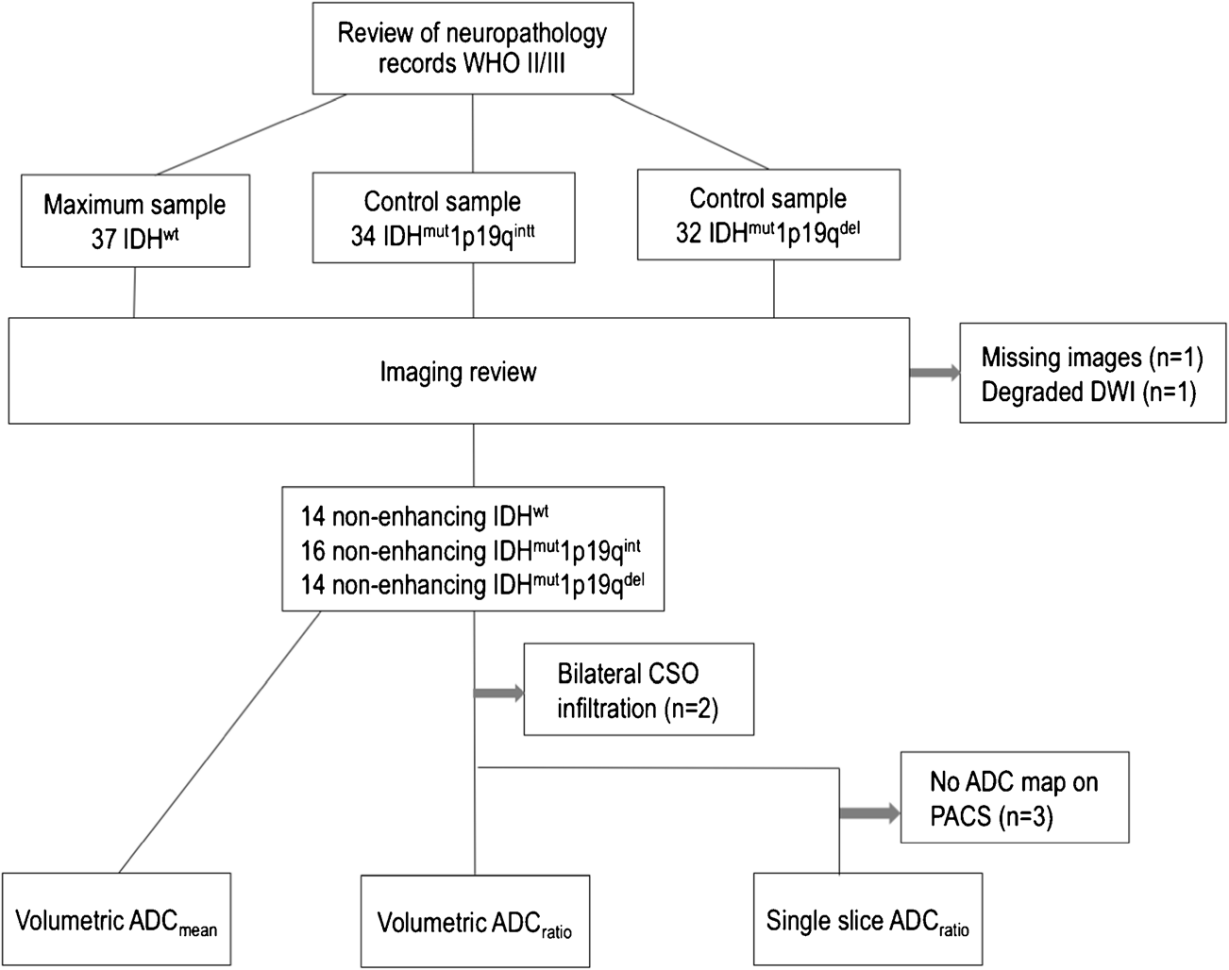
Flow diagram of patients included and excluded from the analyses

Following institutional board approval for a retrospective study, we searched the neuropathology records revealing 37 patients with WHO grade II/III IDH^wt^ glioma between 2009 and 2016. For comparison of the molecular groups, control samples of IDH (IDH1-R132H) mutant gliomas (34 IDH^mut^1p19q^int^ and 32 IDH^mut^1p19q^del^) were randomly selected. We sought to evaluate ADC for suspected LGG prior to tissue diagnosis. To replicate the clinical situation, only gliomas without gadolinium enhancement were included (2 non-enhancing gliomas were excluded because of missing images and degraded DWI, respectively). The study sample consisted of 14 IDH^wt^ (7 WHO II and 7 WHO III), 16 IDH^mut^1p19q^int^ (8 WHO II and 8 WHO III) and 14 IDH^mut^1p19q^del^ (11 WHO II and 3 WHO III), amounting to 44 non-enhancing gliomas for the three molecular groups (patient selection diagram shown in Fig. 1). No haemorrhagic or necrotic gliomas were featured in the study.

### MRI acquisition

**Fig. 2:**
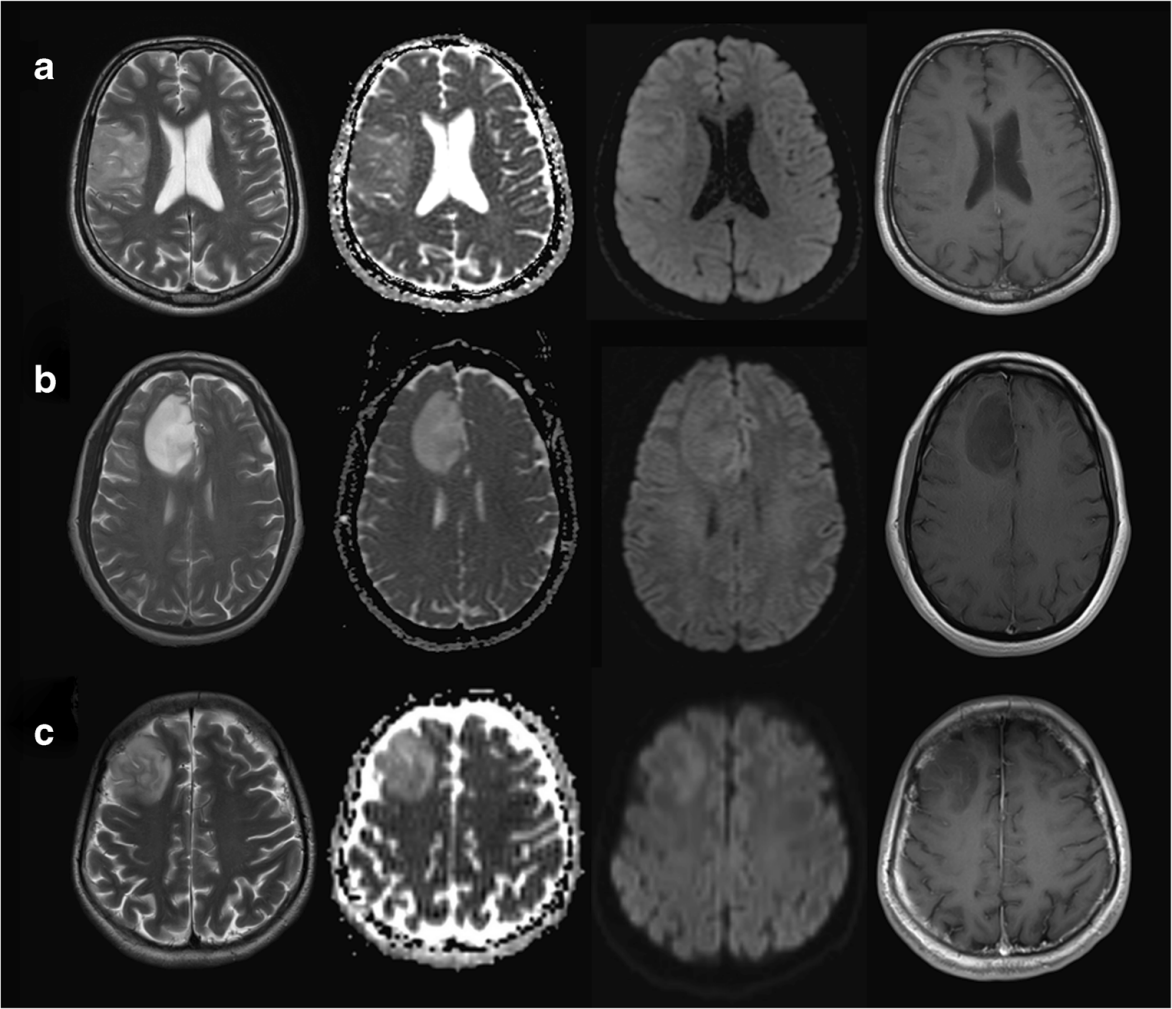
WHO II/III molecular subgroup examples showing T2-weighted images, *b*1000, ADC maps and T1-weighted post gadolinium images of non-enhancing **a** IDH^wt^, **b** IDH^mut^1p19q^int^ and **c** IDH^mut^1p19q^del^ glioma

Ours is a quaternary neurosurgical centre; therefore the standard (structural and DWI) MRI sequences in this study originated from 10 different referring institutions (institution 1 to institution 10): 29 from our own institutions, 4 from institution 2, 3 from institution 3, 2 from institution 4, and one each from the remaining six institutions. The studies were acquired on 18 different scanners (31 at 1.5 Tesla, and 13 at 3 Tesla) from all major vendors: four General Electric scanners [Discovery MR450 (number of patients *n* = 5), 2× Signa Excite (*n* = 1 each), Genesis Signa (*n* = 2)], seven Siemens scanners [3× Avanto (*n* = 7, *n* = 2, *n* = 1), a Trio (*n* = 9), Symphony (*n* = 4), Skyra (*n* = 3), Espree (*n* = 1)], six Philips scanners [Ingenia (*n* = 2), 5× Achieva (*n* = 1 each)] and one Toshiba scanner (*n* = 1). All acquisitions included axial T2-weighted images, and axial standard 3-directional whole brain DWI. The median [min, max] values of the parameters of the T2-weighted images were echo time (TE) = 99.5 [80, 141] ms; repetition time (TR) = 4610 [2500, 7480] ms, in-plane resolution = 0.5 × 0.5 [0.3 × 0.3, 0.9 × 0.9] mm^2^; slice thickness = 5 [1, 6] mm; gap between slices = 1.5 [0, 2] mm. All DWI acquisitions included diffusion gradient weighting values *b* = 0 s/mm^2^ and *b* = 1000 s/mm^2^; the median [min, max] of other parameters were TE = 90.5 [69.5, 137] ms; TR = 4000 [2837, 10,000] ms, in-plane resolution = 1.25 × 1.25 [0.5 × 0.5, 2.5 × 2.5] mm^2^; slice thickness = 5 [4, 6] mm; gap between slices = 1.5 [0, 2] mm. For each patient, the imaging study was performed on average (standard deviation, sd) 2.3 (2.8) months prior to the tissue diagnosis. Image examples for the glioma molecular subgroups are shown in Fig. 2.

### Post-processing and ADC analysis

#### ADC map calculation

In a spin echo diffusion-weighted sequence, the signal *S*_b_ [*S*_b_ = *S*_0_ e^(−*b* ADC)^] from each pixel in an image is formed of a first component (*S*_0_) dependent on tissue properties (i.e. ‘spin density’, T_1_ and T_2_ relaxation times) and sequence properties (e.g. repetition time, TR); and a second component (e^−*b* ADC^) dependent on the diffusion gradients (*b*, in units of s/mm^2^) and the apparent diffusion coefficient (ADC, in units of mm^2^/s).

The ADC is obtained by dividing the image acquired without diffusion gradients (*S*_*b* = 0_ = S_0_) by the image acquired with diffusion gradients (*S*_b_):

1 $$ \mathrm{ADC}=\left(1/b\right)\ \ln \left({S}_0/{S}_{\mathrm{b}}\right) $$

In this division, the dependence of ADC from S_0_ (and therefore from T_1_, T_2_ and TR) is eliminated [26]. The ADC maps were calculated using Eq. 1 and the utility fslmaths from the software library fsl (version 5.0) [27]. Offline whole tumour analysis and single slice analysis were subsequently performed.

### Whole tumour (volumetric) ADC analysis

Tumour volumes of interest (VOI_tum_) were outlined by a neuroradiology resident (S.H.) using ITK snap Toolbox version 3.6 (www.itksnap.org [28]), covering the entire T2 signal abnormality with each segmentation optimised by a board-certified neuroradiologist specialised in brain tumour imaging (S.C.T.). For multicentric gliomas, the total volume of signal abnormality was treated as one lesion. ADC maps were co-registered to T2 imaging using the FLIRT toolbox [29, 30] performing a rigid body transformation with a six-parameter model and ‘Normalised Mutual Information’ as cost function. Subsequently, ADC_mean_ measurements were obtained for each tumour, using the fslstats utility from fsl [25–27].

To consider possible interindividual variations in brain diffusivity, we assessed the ADC_mean_ in normal appearing white matter (NAWM). For each patient, a standardised second volume of interest (VOI_CS_) was drawn in the contralateral centrum semiovale (CS). This VOI_CS_ was used to calculate the ADC_ratio_ = ADC_mean_(VOI_tum_)/ADC_mean_(VOI_CS_) (Fig. 2). For two IDH^wt^ tumours, the NAWM analysis was omitted because of bilateral tumour infiltration.

### Single slice ADC analysis

**Fig. 3:**
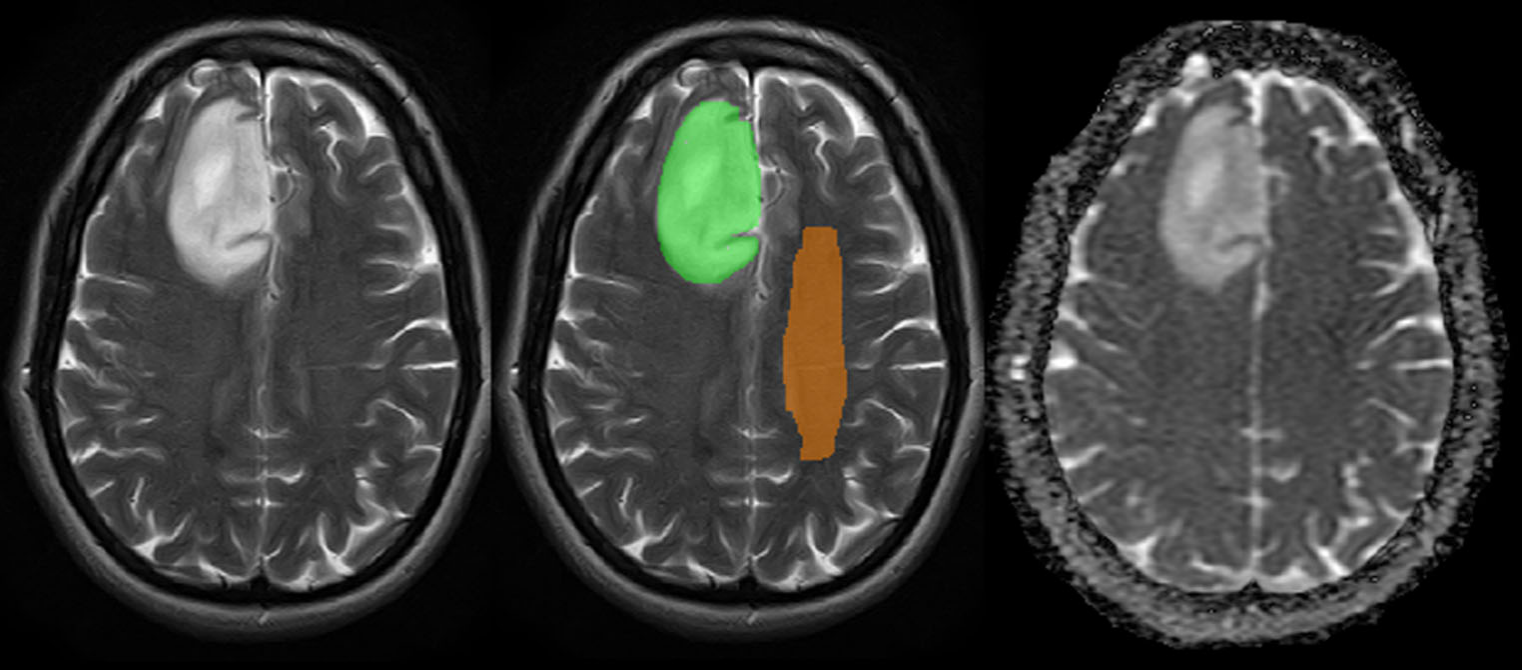
Image examples demonstrating the whole lesion volumetric segmentation (mask overlaid on right frontal IDH^mut^1p19q^int^ glioma), single slice largest tumour cross-section ROI_tum_ and comparative contralateral NAWM ROI_CS_ placements

Standard picture archiving and communication systems (PACS) software (IMPAX 6.5.1.1008, Agfa-Gevaert, Mortsel, Belgium) was used to exploit tools routinely available for reporting of MR images. Two observers blinded to histomolecular results (J.A.M. general radiology trainee = observer 1 and S.C.T. = observer 2) located the tumour on the T2-weighted sequence, selecting two round regions of interest on the ADC map viewed side-by-side: The first region of interest (ROI_tum_) was drawn in the largest lesion cross-section sparing the tumour margin to avoid partial volume effects. The second round ROI_CS_ aiming for a similar size to ROI_tum_ was placed in contralateral centrum semiovale NAWM, taking care to exclude images with visible ventricular surfaces, cortex and/or sulcal spaces at measurement level. Three patients were excluded from the single slice analysis because of non-availability of an ADC map on PACS. The ratio between the ADC_mean_ in the tumour and CS was calculated [PACS_ADC_ratio_ = ADC_mean_(ROI_tum_)/ADC_mean_(ROI_CS_)]. No absolute ADC values were measured by the single slice method, as their workstation display can vary depending on the referring institution. An example of the volumetric segmentation and single slice ADC measurement is demonstrated in Fig. 3.

### Histopathology and molecular analysis

Paraffin blocks containing tissue were analysed at our institution’s neuropathology department according to WHO 2016 guidance and previously published data [16]. IDH R132H immuno-negative tumours underwent multiple gene Sanger sequencing. A quantitative polymerase chain reaction-based copy number assay was used to determine 1p/19q status.

### Statistical analysis

For the volumetric and single slice data, the statistical analysis consisted of two steps each: (i) linear regression to assess the association between the tumour type (IDH^wt^, IDH^mut^1p19q^int^, IDH^mut^1p19q^del^) and ADC values, followed by (ii) logistic regression to determine if ADC values can differentiate IDH^wt^ from IDH^mut^ gliomas. A receiver operating characteristic (ROC) analysis was used to quantify the performance of the logistic regression. For the identification of a cut-off point for the logistic regression the ‘nearest to (0,1)’ method was performed. Statistical significance was set at 5%. The inter-rater agreement was expressed as an intraclass correlation coefficient (ICC) using a two-way random effects model. All statistical analyses were performed using Stata version 14 (College Station, TX: StataCorp LP).

## Results

The mean age was greater in the IDH^wt^ group than in the IDH^mut^ groups (*p* = 0.0001 for IDH^mut^1p19q^int^, *p* = 0.005 for IDH^mut^1p19q^del^). The larger proportion of WHO II gliomas in the IDH^mut^1p19q^del^ was not statistically significant (Pearson chi-square test *p* = 0.115 for IDH^wt^ and *p* = 0.105 for IDH^mut^1p19q^int^). The patient demographic data and tumour volumes are reported in Table 1.

**Table 1:** Patient demographic data and tumour volumes

Whole tumour ADC_mean_ (VOI_tum_)					
Patient group	Nr of patients total (male)	Age in years (mean ± sd) (years)	Tumour volume (mean ± sd) (cm^3^)	CS NAWM volume (mean ± sd) (cm^3^)	Tumour volume for patients with bilateral infiltration (mean ± sd) (cm^3^)
IDH^wt^	14 (9)	53 (± 14)	64 (± 68) (*n* = 12)	11.6 (± 2.5) (*n* = 12)	366 (± 46) (*n* = 2)
IDH^mut^1p19^int^	16 (6)	33.9 (± 8.6)	60 (± 44)	10.9 (± 2.3)	N/A
IDH^mut^1p19^del^	14 (7)	38.9 (± 8.3)	48 (± 50)	10.8 (± 2.5)	N/A

### Association between molecular subtype and ADC values

In the volumetric analysis, IDH^wt^ tumours showed significantly lower whole tumour volume ADC_mean_(VOI_tum_) than IDH^mut^1p19q^int^ (*p* < 0.0005) and IDH^mut^1p19q^del^ (*p* = 0.001). The ADC_mean_(VOI_tum_) in the IDH^mut^1p19q^int^ group was significantly higher than in the IDH^mut^1p19q^del^ group (*p* = 0.0047).

IDH^wt^ gliomas had a significantly lower whole tumour ADC_ratio_ than IDH^mut^1p19q^int^ (*p* < 0.0005) and IDH^mut^1p19q^del^ (*p* = 0.019). The ADC_ratio_ in the IDH^mut^1p19q^int^ group was significantly higher than in the IDH^mut^1p19q^del^ group (*p* = 0.0054).

On single slice assessment, a significantly lower mean PACS_ADC_ratio_ was observed for IDH^wt^ than for IDH^mut^1p19q^int^ (*p* < 0.0005 observer 1; *p* < 0.0005 observer 2) and for IDH^mut^1p19q^del^ (*p* = 0.001 observer 1; *p* = 0.001 observer 2). The PACS_ADC_ratio_ in the IDH^mut^1p19q^int^ group was higher than in the IDH^mut^1p19q^del^ group (*p* = 0.0008 for observer 1 and *p* = 0.0025 for observer 2). No statistical associations were demonstrated between the NAWM ADC_mean_ values and molecular subtype.

**Fig. 4:**
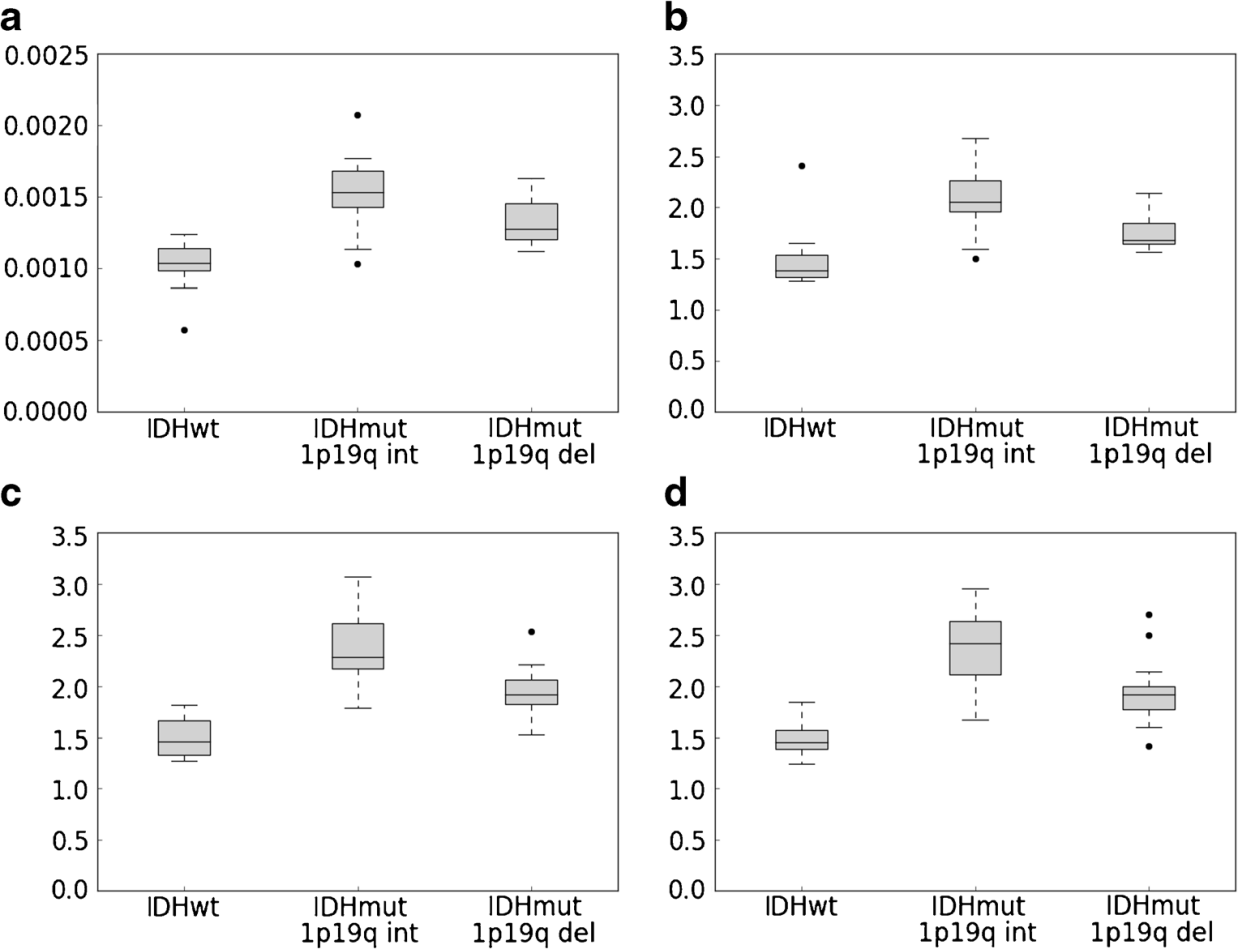
Boxplot of the values of the **a** whole tumour ADC_mean_(VOI_tum_), **b** whole tumour ADC_ratio_, **c** single slice PACS_ADC_ratio_ first observer and **d** single slice PACS_ADC_ratio_ first observer

The intra-rater agreement for the PACS_ADC_ratio_ measurements was very high: the correlation of measurements made on the same individual was 0.96, while the correlation between mean observer ratings was 0.98. The correlation of measurements equaled the consistency agreement, indicating no systematic difference between the two observers. The single slice ADC_ratio_ values were slightly but systematically higher than the volumetric ADC_ratio_. The numerical results of the association between tumour type and ADC values for the volumetric and single slice analyses are reported in Table 2. In Table 3, the difference between the ADC values in IDH^mut^1p19q^int^ and in IDH^mut^1p19q^del^ is shown. In Table 4 the ICC values are detailed. The boxplots of the ADC_mean_ and ADC_ratio_ values are depicted in Fig. 4.

**Table 2:** Results of the linear regression between ADC and tumour type (IDH^wt^ is the reference group)

Whole tumour ADC_mean_ (VOI_tum_)				
Patient group	ADC_mean_(VOI_tum_) mean (sd) (10^−6^ mm^2^/s)	Regression coefficient (10^−6^ mm^2^/s)	95% CI of the regr. coeff. (10^−6^ mm^2^/s)	*p*
IDH^wt^	1032 (168)	1032	922–1141	0.0005
IDH^mut^1p19^int^	1543 (254)	511	361–661	0.0005
IDH^mut^1p19^del^	1321 (162)	289	134–444	0.001
Whole tumour ADC_ratio_				
Patient group	ADC_ratio_ mean (sd)	Regression coefficient	95% CI of the regr. coeff.	*p*
IDH^wt^	1.49 (0.32)	1.49	1.32–1.66	0.0005
IDH^mut^1p19^int^	2.09 (0.34)	0.59	0.37–0.82	0.0005
IDH^mut^1p19^del^	1.77 (0.20)	0.28	0.05–0.51	0.019
Single slice PACS_ADC_ratio_ first observer				
Patient group	PACS_ADC_ratio_ mean (sd)	Regression coefficient	95% CI of the regr. coeff.	*p*
IDH^wt^	1.50 (0.21)	1.50	1.33–1.68	0.0005
IDH^mut^1p19^int^	2.37 (0.35)	0.87	0.63–1.10	0.0005
IDH^mut^1p19^del^	1.96 (0.27)	0.45	0.20–0.70	0.001
Single slice PACS_ADC_ratio_ second observer				
Patient group	PACS_ADC_ratio_ mean (sd)	Regression coefficient	95% CI of the regr. coeff.	*p*
IDH^wt^	1.48 (0.19)	1.48	1.28–1.68	0.0005
IDH^mut^1p19^int^	2.37 (0.38)	0.88	0.62–1.14	0.0005
IDH^mut^1p19^del^	1.96 (0.36)	0.47	0.20–0.75	0.001

Regression coefficient represents the difference in the dependent variable (ADC) between each of the two IDH^mut^ groups and the reference group (IDH^wt^)

**Table 3:** *F* test for the difference between IDH^mut^1p19^int^ and IDH^mut^1p19^del^

Analysis type	*p*
ADC_mean_ (VOI_tum_)	0.0047
Whole tumour ADC_ratio_	0.0054
PACS_ADC_ratio_ 1st observer	0.0008
PACS_ADC_ratio_ 2nd observer	0.0025

**Table 4:** Inter-rater agreement expressed as intraclass correlation coefficient (ICC)

	Correlation ICC (95% CI)	Consistency ICC (95% CI)
Observer 1 vs observer 2 - PACS_ADC_ratio_		
Individual ICC	0.96 (0.92–0.98)	0.96 (0.92–0.98)
Average ICC	0.98 (0.96–0.99)	0.98 (0.96–0.99)
Observer 1 PACS_ADC_ratio_ vs volumetric ADC_ratio_		
Individual ICC	0.80 (0.35–0.92)	0.87 (0.77–0.93)
Average ICC	0.89 (0.52–0.96)	0.93 (0.87–0.96)
Observer 2 PACS_ADC_ratio_ vs volumetric ADC_ratio_		
Individual ICC	0.79 (0.43–0.91)	0.85 (0.74–0.92)
Average ICC	0.88 (0.60–0.95)	0.92 (0.85–0.96)

### Diagnostic performance of ADC values

For ADC_mean_(VOI_tum_), a ROC analysis quantified the accuracy of correctly classifying tumour type to an area under the curve (AUC) of 0.94. The cut-off point for the ADC_mean_(VOI_tum_) was 1201 × 10^−6^ mm^2^/s, with a sensitivity of 0.83 and a specificity of 0.86. For a decrease in the ADC_mean_(VOI_tum_) value by 1.0 × 10^−5^ mm^2^/s, the odds of IDH^wt^ increased by 78% (*p* = 0.003).

For the volumetric ADC_ratio_, the ROC analysis yielded an AUC of 0.90 with a sensitivity of 0.80 and a specificity of 0.92 for a threshold ADC_ratio_ of 1.65. For a decrease in the volumetric ADC_ratio_ value by 0.1, the odds of IDH^wt^ increased by 46% (*p* = 0.004).

**Fig. 5:**
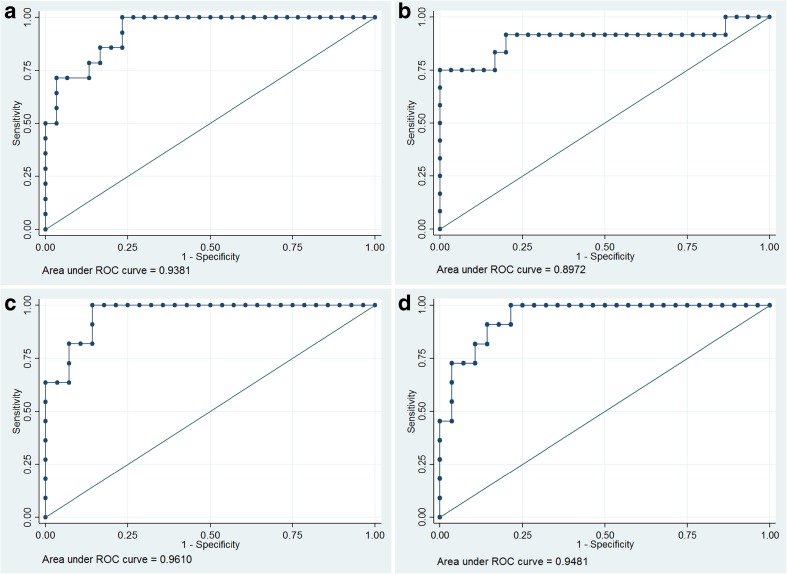
ROC curves for the **a** whole tumour ADC_mean_(VOI_tum_), **b** whole tumour ADC_ratio_, **c** single slice PACS_ADC_ratio_ observer 1 and **d** observer 2

A ROC analysis quantified the accuracy of the PACS_ADC_ratio_ logistic regression in correctly classifying tumour type to an AUC of 0.96 for observer 1 and 0.95 for observer 2. The cut-off point for the PACS_ADC_ratio_ for observer 1 (observer 2) was 1.83 (1.76) with a sensitivity of 0.80 (0.86) and a specificity of 1.00 (0.91) at the cut-off point. For a decrease in the single slice ADC_ratio_ value by 0.1, the odds of IDH^wt^ increased by 62% (*p* = 0.005) for observer 1 and 57% (*p* = 0.004) for observer 2. The numerical results for glioma subtype prediction are reported in Table 5. The ROC curves are depicted in Fig. 5.

**Table 5:** Cut-off point estimation

Method	Cut-off point	Sensitivity at cut-off point	Specificity at cut-off point	AUC at cut-off point
ADC_mean_(VOI_tum_)	1201 (10^−6^ mm^2^/s)	0.83	0.86	0.85
Whole tumour ADC_ratio_	1.65	0.80	0.92	0.86
PACS_ADC_ratio_ 1st observer	1.83	0.86	1.00	0.93
PACS_ADC_ratio_ 2nd observer	1.76	0.86	0.91	0.88

## Discussion

In this analysis, we observed that ADC values obtained from standard clinical DWI are a highly significant predictor of non-enhancing glioma IDH status and may permit non-invasive molecular subtyping in accordance with the 2016 WHO classification.

Two recent surveys highlighted clinical practices in caring for patients with presumed LGG, with approximately 50% of neurosurgeons adopting a ‘wait and see’ approach balanced against surgical risk [31], and only 21% performing an upfront biopsy [32]. Consequently, innocuous appearing IDH^wt^ gliomas may reveal their aggressive nature through progression and receive treatment with a delay.

Low ADC values are associated with increased glioma cellularity and worse prognosis, supported by comparisons of diffusivity, histological specimens and clinical data in multiple studies [5, 33–37]. Low diffusivity predicts poor astrocytoma survival independent from WHO grade [38], although no linear relation exists between ADC and glioma prognosis [39].

Past studies to distinguish astrocytoma and oligodendroglioma using ADC values yielded variable success [40, 41], and in retrospect may have been influenced by the incomplete overlap between histological and molecular groups. Diagnostic focus has shifted to genetic typing, yet immunohistochemistry tests are complex and not infallible, requiring interpretation in in the context of morphological criteria and test type performed to avoid interpretational errors [42].

Recently, Leu et al. were able to assign gliomas to the WHO 2016 molecular groups using ADC; however, their method differed from ours by including enhancing lesions and ADC median values derived from *b*700–1000 gradients with DTI analysed for some patients [43]. To our best knowledge, this is the first IDH typing study to focus on non-enhancing gliomas, using *b*1000 values derived from 3-directional DWI. This is particularly important, as such tumours are usually assumed to be less aggressive in common clinical practice.

We found ADC_ratio_ values to be closely reproducible when comparing whole lesion measurements against single slice region of interest placements, for which there was near complete interobserver agreement. The similarity of our volumetric and single slice results could be explained by a relative homogeneity of these non-enhancing, non-necrotic gliomas. Both the absolute ADC_mean_ values and ADC_ratio_ appear valuable for this lesion type. The quicker and easier single slice analysis even performed marginally better. This is in line with results of previous imaging research, which suggested that whole lesion diffusivity measurement is not always superior to ROI analysis [25, 44].

The ability of ADC to predict glioma subtypes and optimum thresholds may be subject to ROI placement technique with previous research focusing on minimum ADC value analysis: Xing et al. showed a statistical correlation between ADC and IDH status using a multiple (≥ 5) ROI technique with the mean of the lowest ADC measurement chosen as minimum ADC in consensus [45]. In a similar fashion, a previous DTI study for IDH typing used multiple ROI placements and a two-reader consensus method to obtain minimum ADC values [24].

As a reference ROI, we chose the centrum semiovale for its potentially greater reproducibility compared to a ‘mirror’ ROI [45], because this could be influenced by tumour location. We avoided the internal capsule [24], which is a smaller structure and more difficult to locate by an untrained rater.

Lee et al. found ADC mean and ADC histograms useful for IDH typing of WHO grade III and IV gliomas [46]. However, for glioblastoma IDH typing alone, a recent study identified no difference in ADC values [47]. In Tan et al.’s study of grade II–IV gliomas, the accuracy of ADC for IDH typing decreased with higher grade, which may reflect greater lesion heterogeneity [24]. It is probable that in such circumstances advanced diffusion acquisitions (e.g. DKI or multi-*b*-value imaging) could provide greater tissue microstructural information.

The good performance of the single slice ROI technique in IDH typing of non-enhancing lower grade gliomas was unexpected, but is highly relevant. It implies that such easy-to-perform measurements could be incorporated into clinical reports, complementing advanced MR modalities such as perfusion and 2HG spectroscopy [48, 49] pending tissue diagnosis. The origin of data from 18 MRI systems could represent a limitation of this study, but reflects clinical reality. The fact that significant separation of glioma subtypes could be obtained from this dataset further underscores the robustness of ADC.

It remains unknown why intermediate ADC values were observed in the 1p19q co-deleted gliomas, despite their best prognosis. This result is consistent with published data on intermediate diffusivity in oligodendroglioma; interestingly this tumour subtype may also mimic malignant gliomas on MR perfusion studies [39, 50].

In summary, the results from this study suggest that for newly diagnosed non-enhancing gliomas with ADC ratio values of 1.8 or less, further investigation with consideration of early tissue diagnosis is advisable given an increased risk of IDH^wt^ molecular status.

## Conclusions

ADC measurement appears to be a simple and powerful method for molecular subtyping of non-enhancing WHO II–III gliomas, specifically to identify IDH^wt^ neoplasms. In our patient cohort, a two-dimensional ROI measurement in the largest lesion cross-section appeared representative of the entire tumour with comparable results.

## Abbreviations

ADC: Apparent diffusion coefficient
AUC: Area under the curve
CS: Centrum semiovale
DKI: Diffusion kurtosis imaging
DTI: Diffusion tensor imaging
DWI: Diffusion-weighted imaging
IDH: Isocitrate dehydrogenase
IDH^wt^: Isocitrate dehydrogenase wild-type
IDH^mut^1p19^int^: Isocitrate dehydrogenase-mutant 1p19q intact
IDH^mut^1p19^del^: Isocitrate dehydrogenase-mutant 1p19q co-deleted
ICC: Intraclass correlation coefficient
LGG: Low grade glioma
NAWM: Normal appearing white matter
PACS: Picture archiving and communications system
ROC: Receiver operating characteristic
ROI_CS_: Centrum semiovale region of interest
ROI_tum_: Tumour region of interest
TE: Echo time
TR: Repetition time
VOI_CS_: Centrum semiovale volume of interest
VOI_tum_: Tumour volume of interest
WHO: World Health Organization
2HG: D2-hydroxyglutarate
